# Supplementary figures and images for: Optical Genome Mapping as a Potential Routine Clinical Diagnostic Method
Source: Genes (Basel). 2024 Mar 7;15(3):342. doi: 10.3390/genes15030342 (PMC10970541; doi:10.3390/genes15030342)

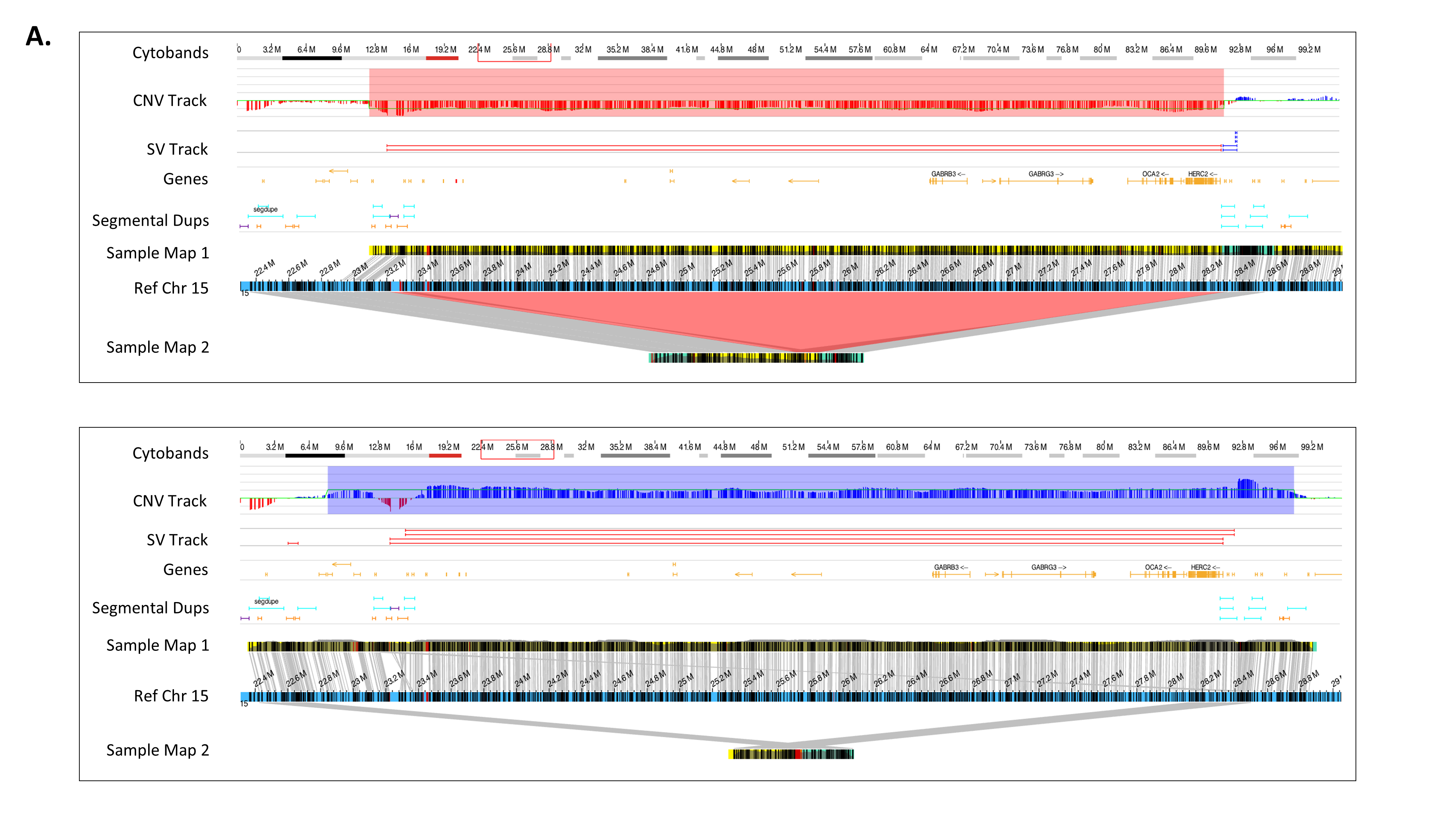

Supplement: Supplementary file 1 [file genes-15-00342-s001.zip › SupplementaryFiles/Supplementary Figure S1. Chromosomal abnormalities detected by OGM/Supplementary Figure S1A.TIF]

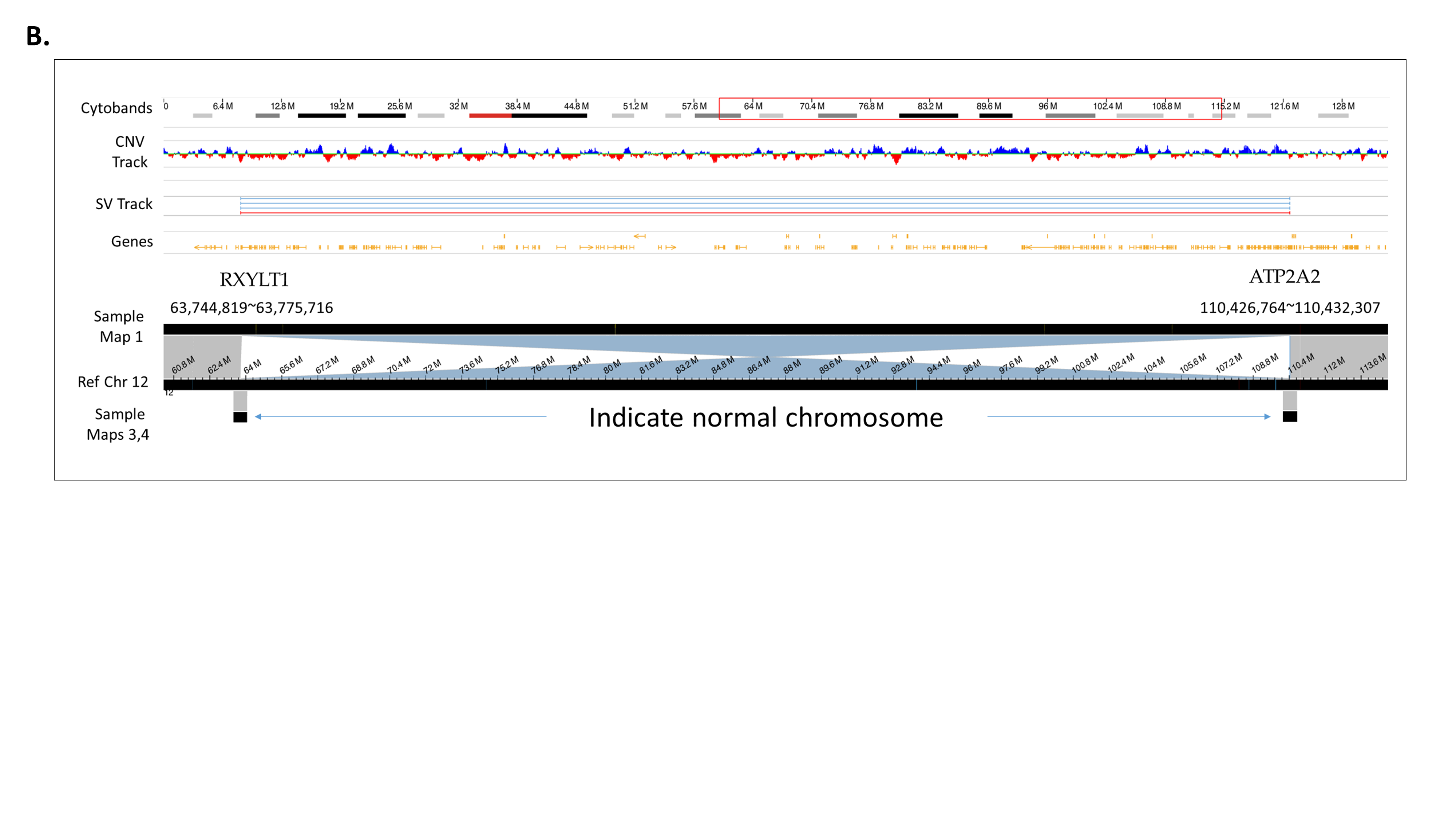

Supplement: Supplementary file 1 [file genes-15-00342-s001.zip › SupplementaryFiles/Supplementary Figure S1. Chromosomal abnormalities detected by OGM/Supplementary Figure S1B.TIF]

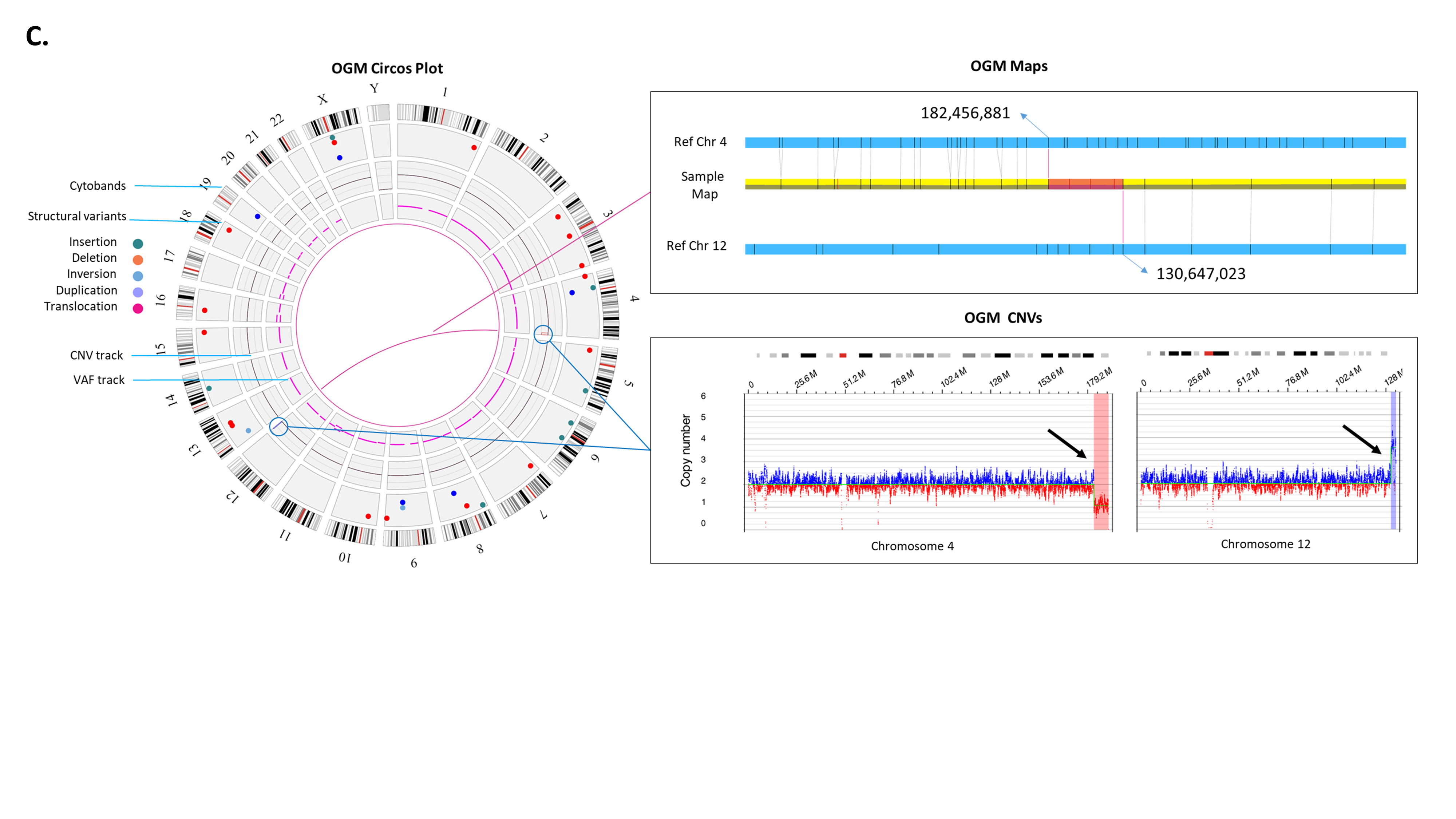

Supplement: Supplementary file 1 [file genes-15-00342-s001.zip › SupplementaryFiles/Supplementary Figure S1. Chromosomal abnormalities detected by OGM/Supplementary Figure S1C.TIF]

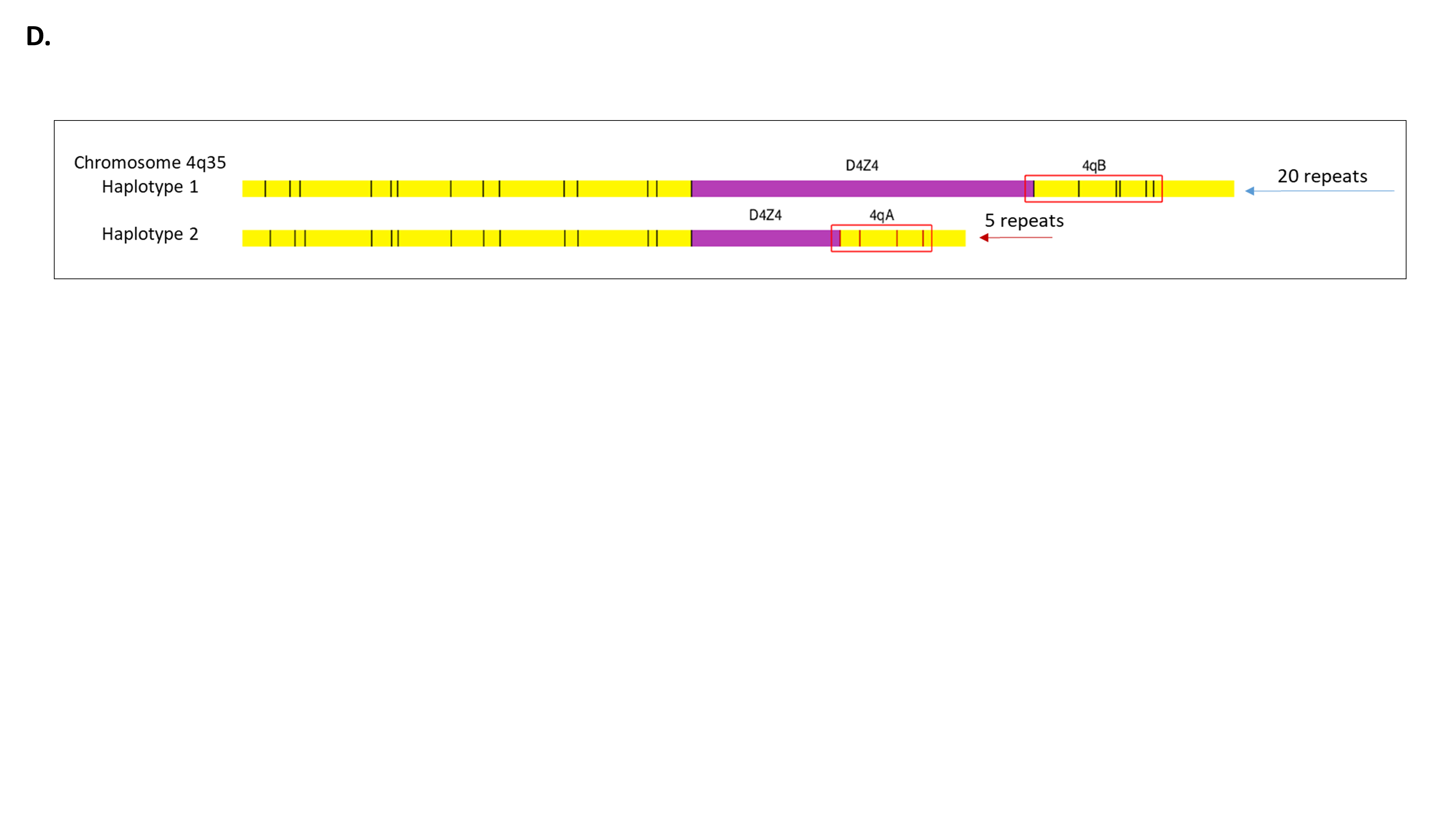

Supplement: Supplementary file 1 [file genes-15-00342-s001.zip › SupplementaryFiles/Supplementary Figure S1. Chromosomal abnormalities detected by OGM/Supplementary Figure S1D.TIF]

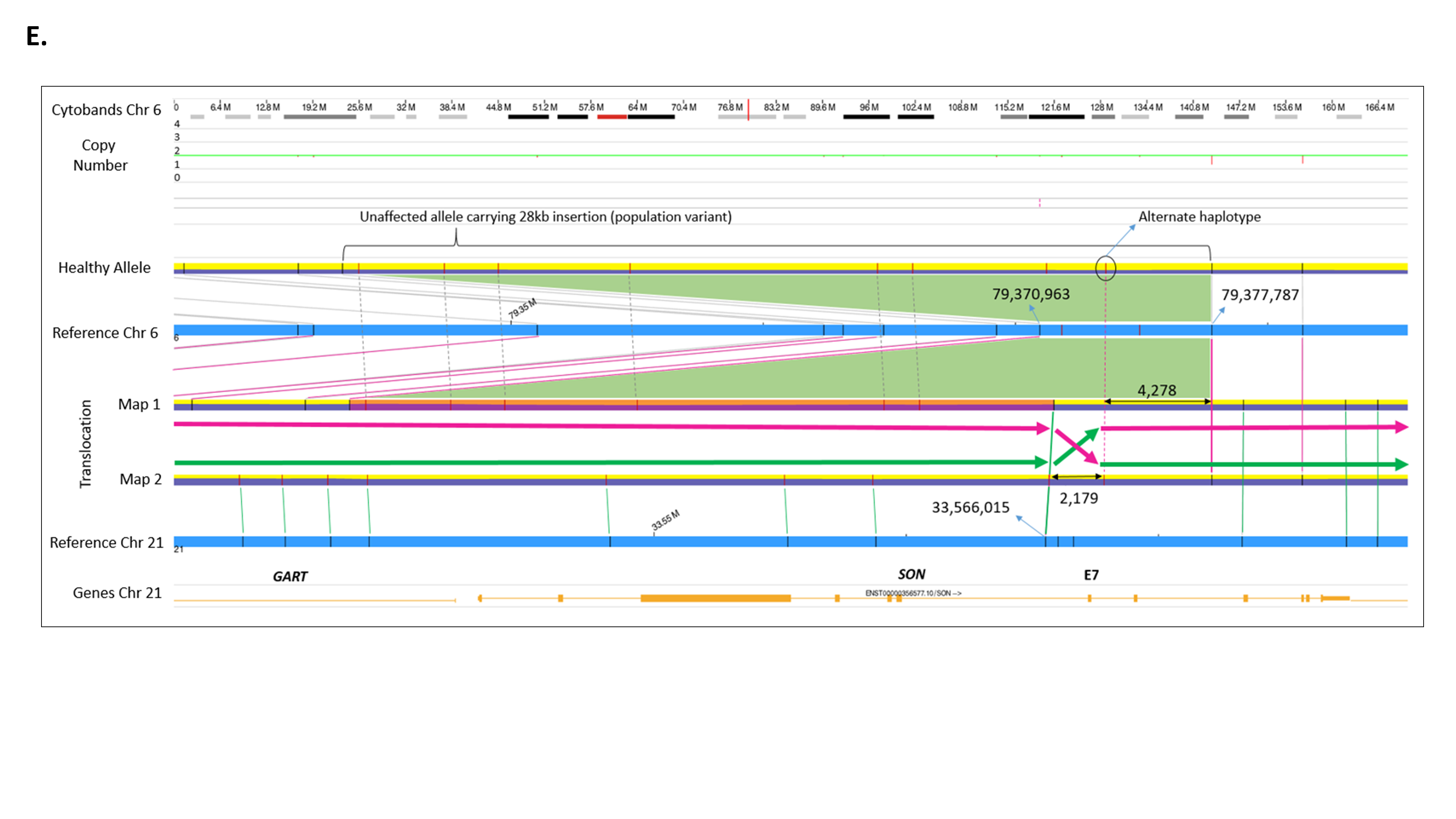

Supplement: Supplementary file 1 [file genes-15-00342-s001.zip › SupplementaryFiles/Supplementary Figure S1. Chromosomal abnormalities detected by OGM/Supplementary Figure S1E.TIF]

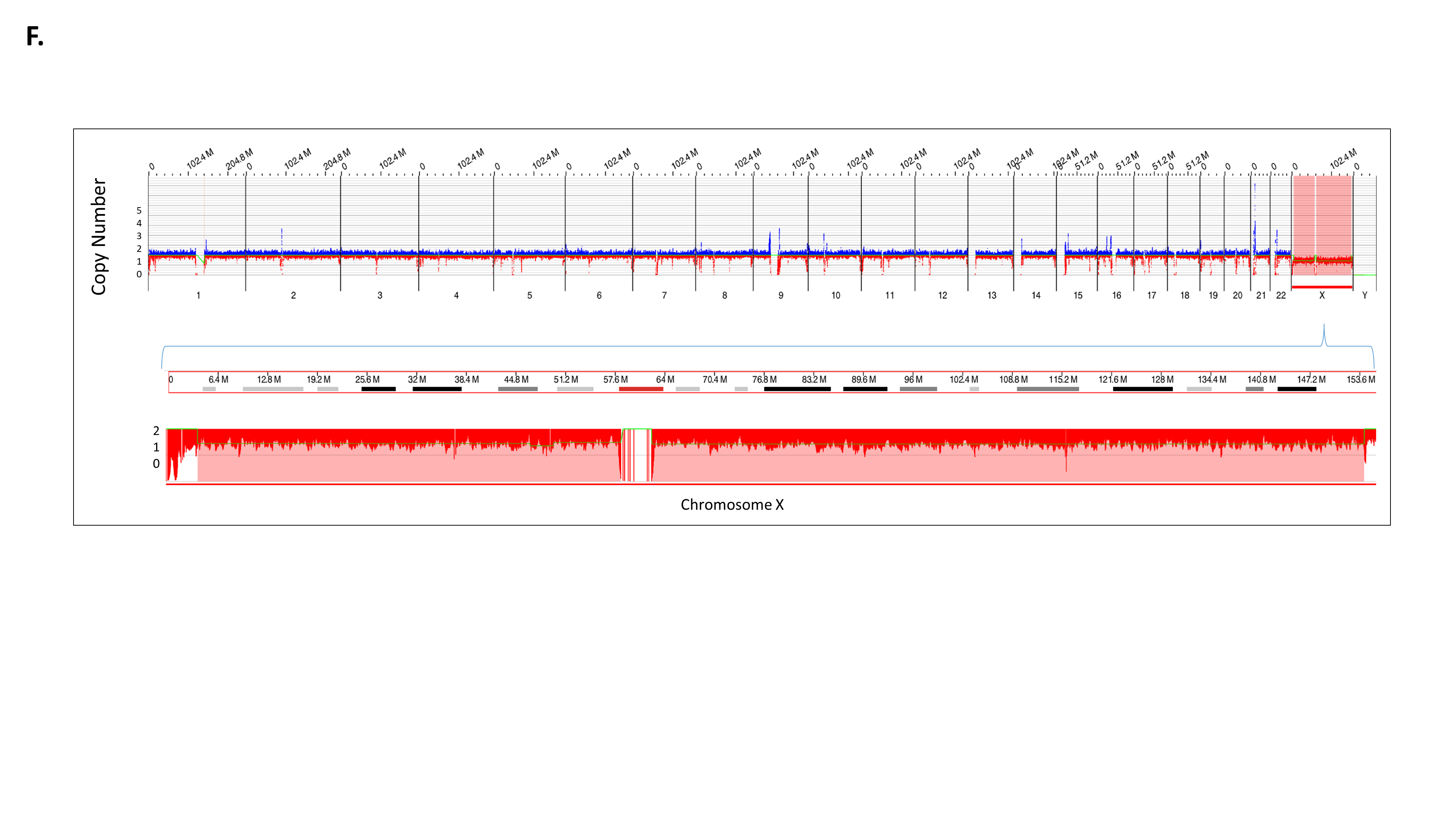

Supplement: Supplementary file 1 [file genes-15-00342-s001.zip › SupplementaryFiles/Supplementary Figure S1. Chromosomal abnormalities detected by OGM/Supplementary Figure S1F.TIF]

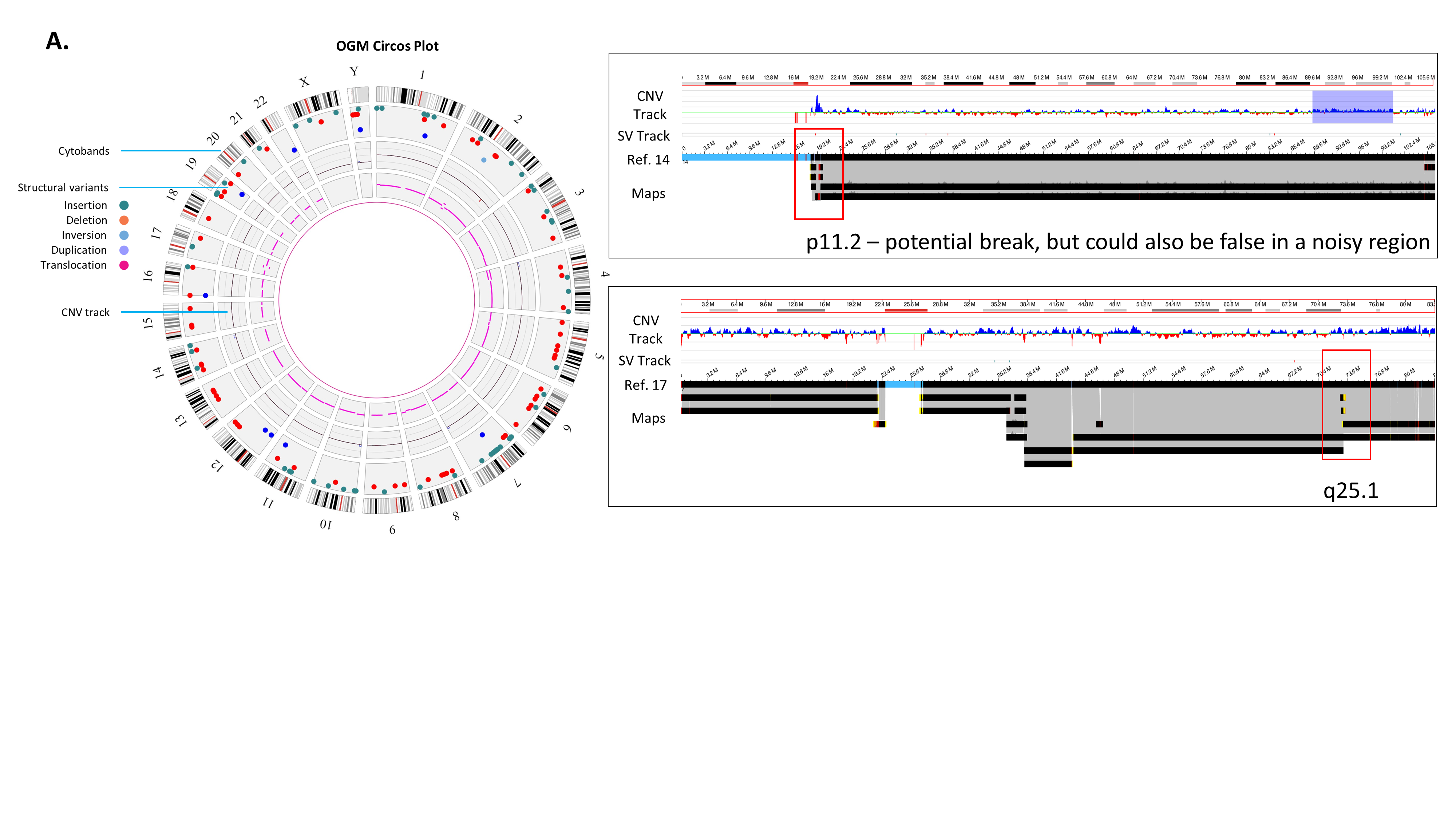

Supplement: Supplementary file 1 [file genes-15-00342-s001.zip › SupplementaryFiles/Supplementary Figure S2. Discordant and ambiguous cases/Supplementary Figure S2A.TIF]

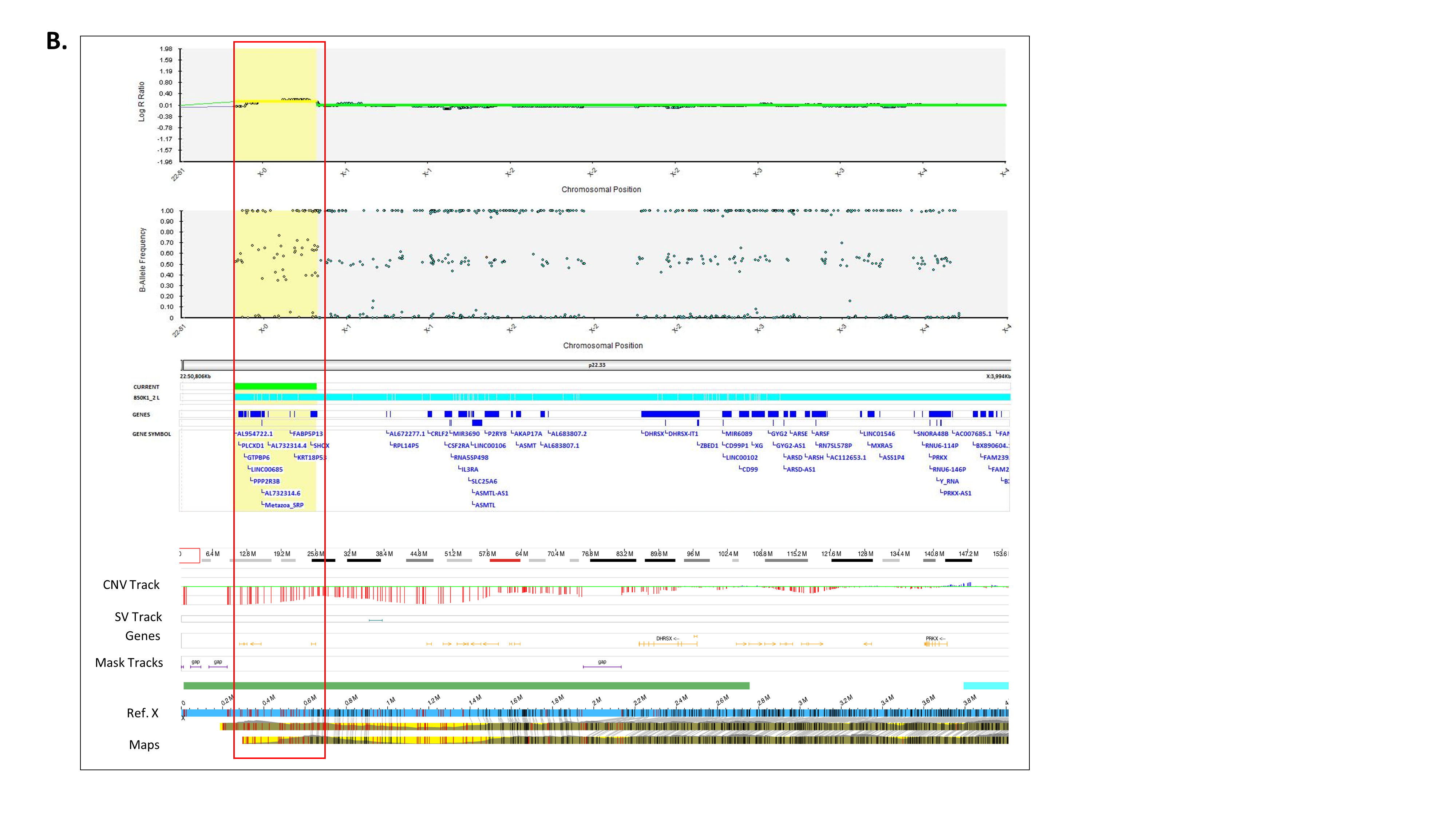

Supplement: Supplementary file 1 [file genes-15-00342-s001.zip › SupplementaryFiles/Supplementary Figure S2. Discordant and ambiguous cases/Supplementary Figure S2B.TIF]

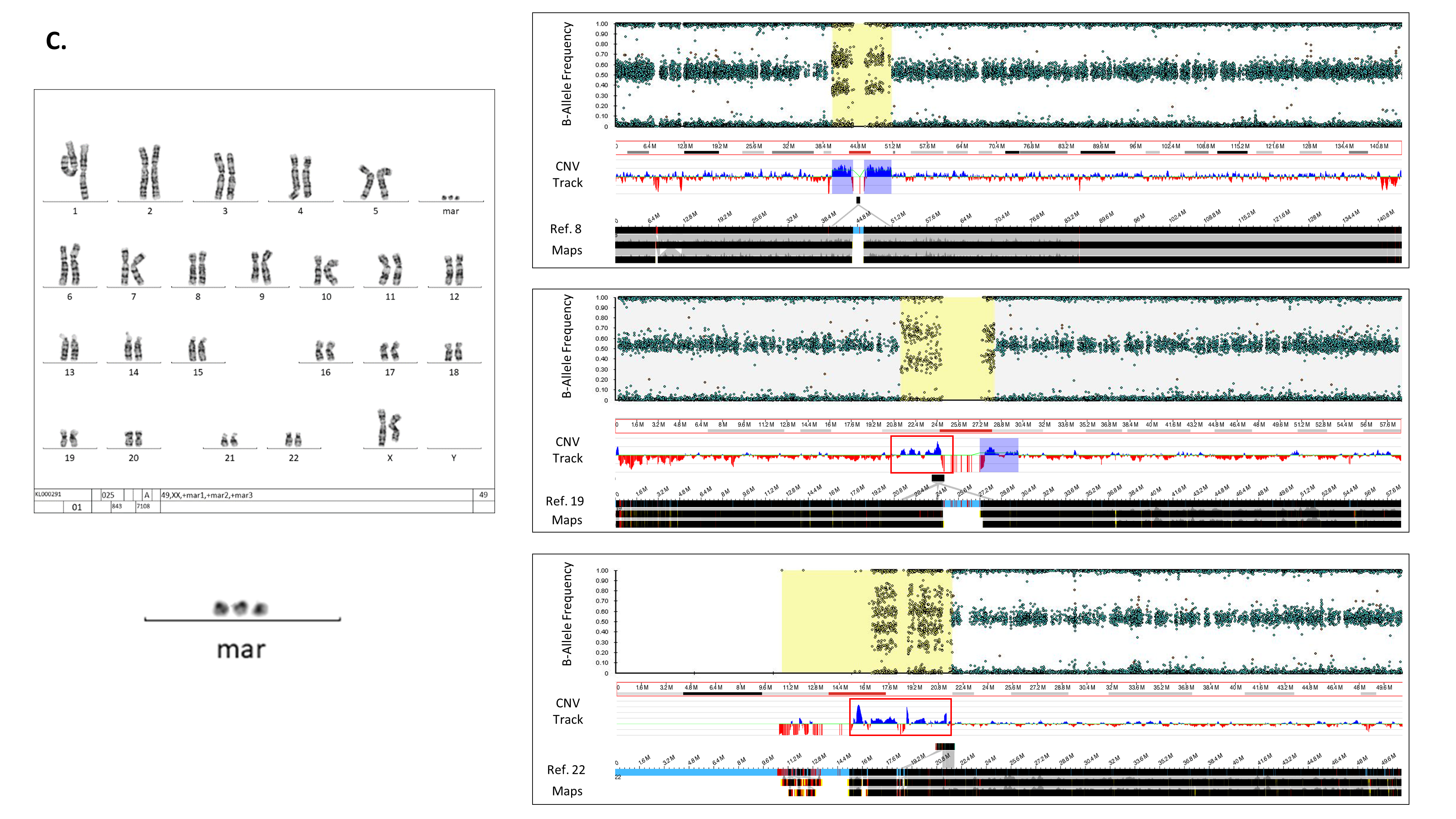

Supplement: Supplementary file 1 [file genes-15-00342-s001.zip › SupplementaryFiles/Supplementary Figure S2. Discordant and ambiguous cases/Supplementary Figure S2C.TIF]

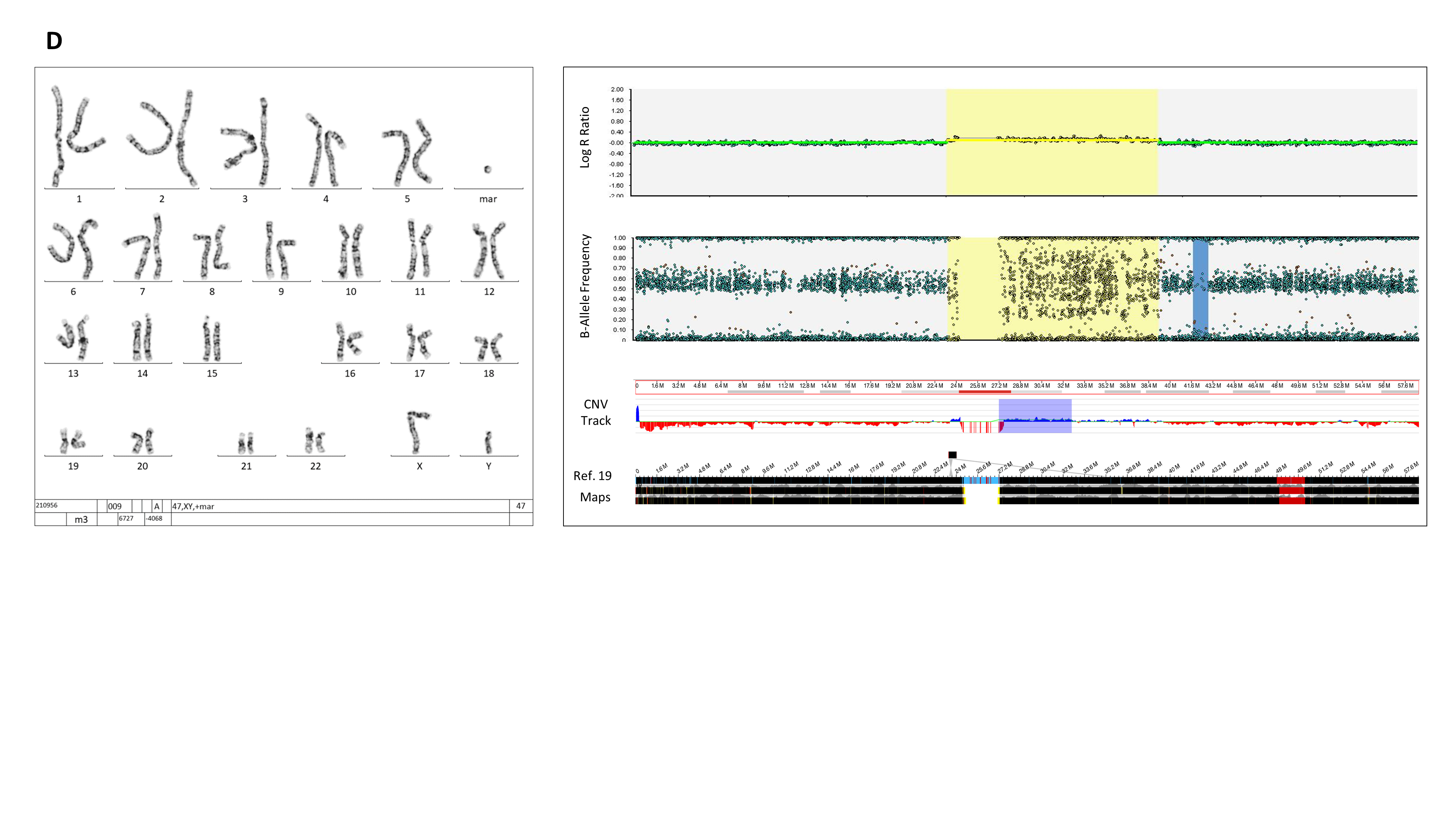

Supplement: Supplementary file 1 [file genes-15-00342-s001.zip › SupplementaryFiles/Supplementary Figure S2. Discordant and ambiguous cases/Supplementary Figure S2D.TIF]

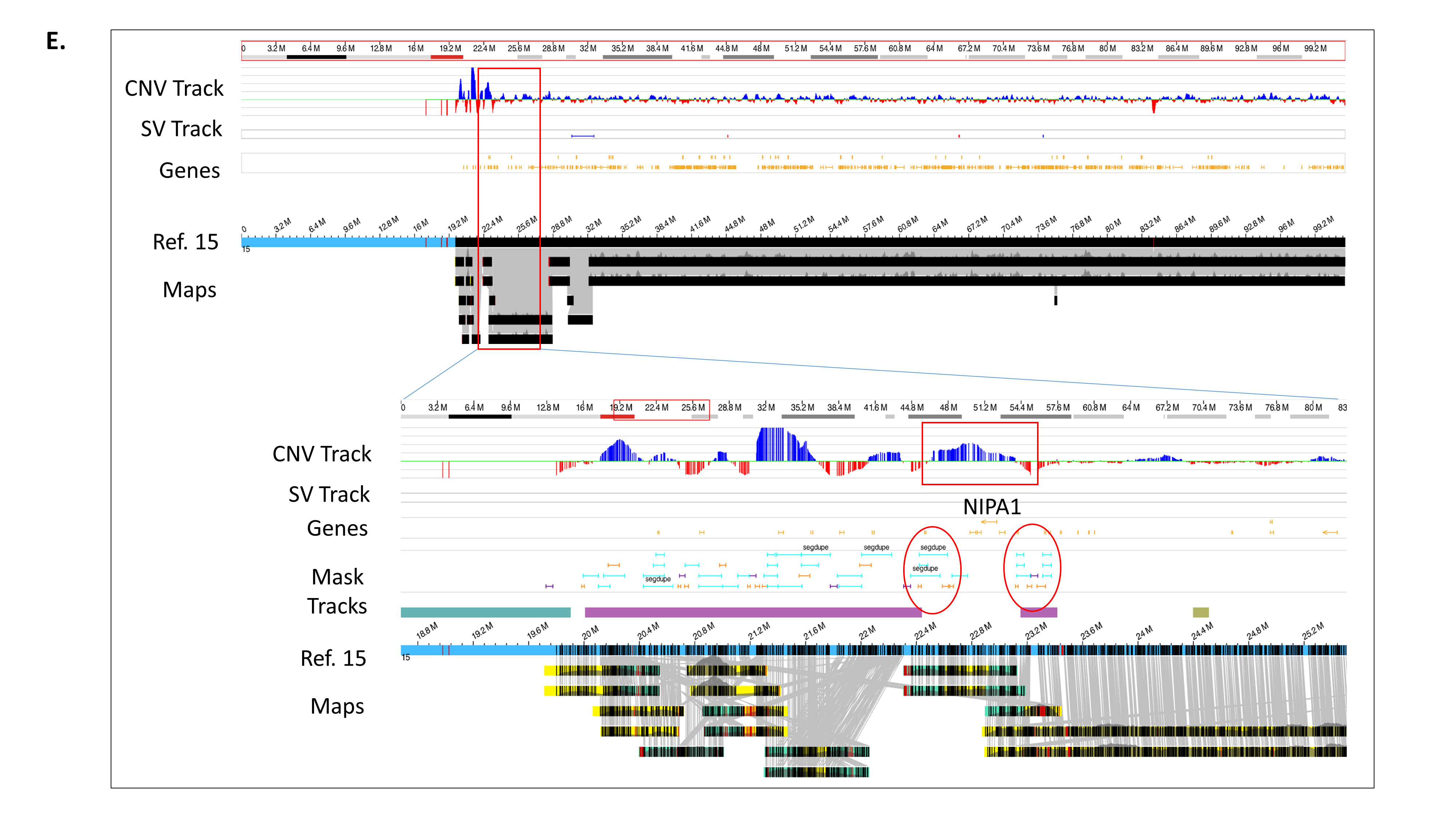

Supplement: Supplementary file 1 [file genes-15-00342-s001.zip › SupplementaryFiles/Supplementary Figure S2. Discordant and ambiguous cases/Supplementary Figure S2E.TIF]

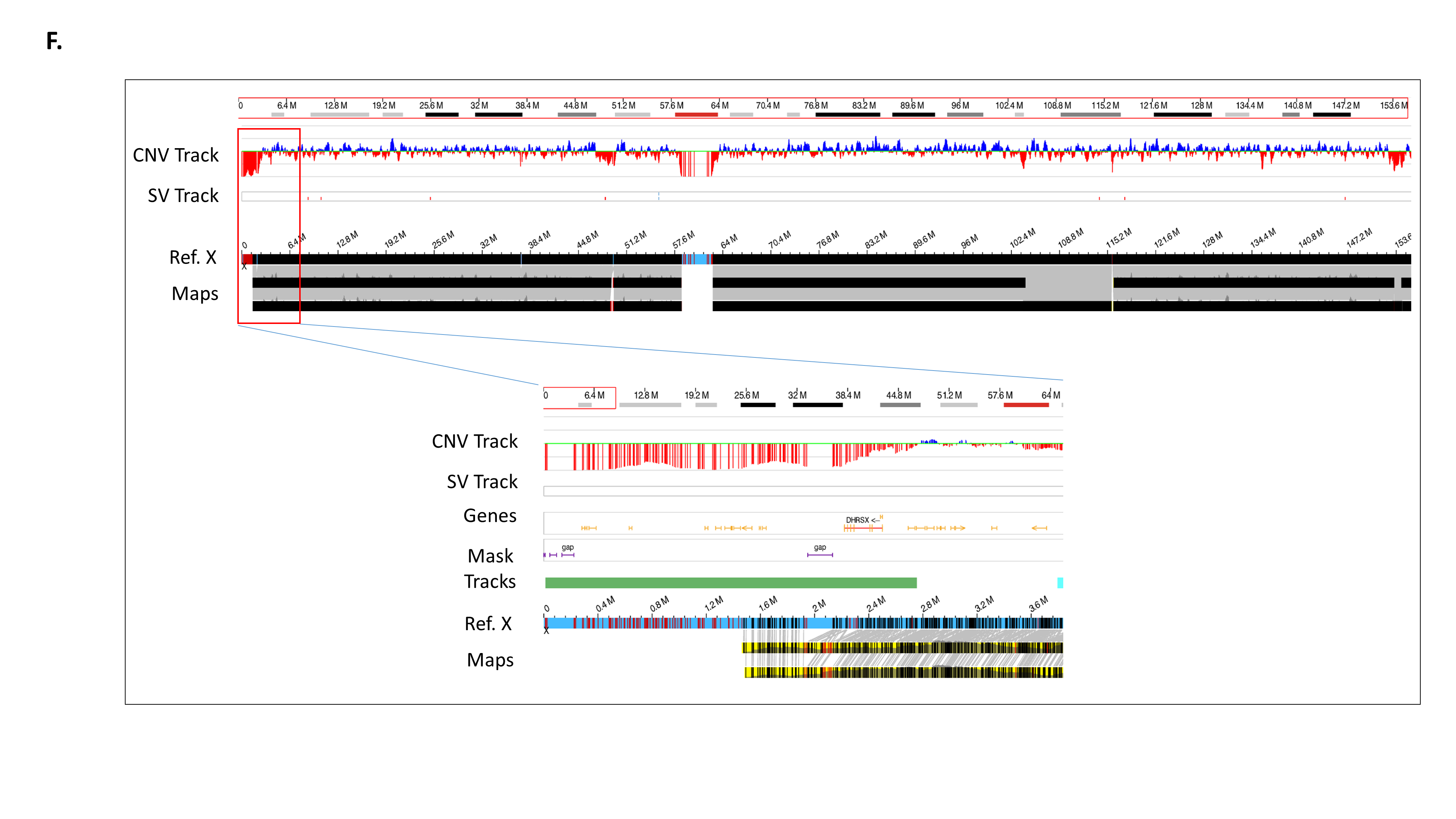

Supplement: Supplementary file 1 [file genes-15-00342-s001.zip › SupplementaryFiles/Supplementary Figure S2. Discordant and ambiguous cases/Supplementary Figure S2F.TIF]
